# Beet Molasses Enhance Salinity Tolerance in *Thymus serpyllum*—A Study under Greenhouse Condition

**DOI:** 10.3390/plants10091819

**Published:** 2021-08-31

**Authors:** Aleksandra Koźmińska, Ewa Hanus-Fajerska, Wiktor Halecki, Krystyna Ciarkowska

**Affiliations:** 1 Department of Botany, Physiology and Plant Protection, Faculty of Biotechnology and Horticulture, University of Agriculture in Kraków, Al. 29 Listopada 54, 31-425 Kraków, Poland; 2 Department of Hydrology, Meteorology and Water Management, Warsaw University of Life Sciences, Nowoursynowska Street 166, 02-787 Warsaw, Poland; wiktor_halecki@sggw.edu.pl; 3 Department of Soil Science and Agrophysics, University of Agriculture in Kraków, Al. Mickiewicza 21, 12-120 Kraków, Poland; krystyna.ciarkowska@urk.edu.pl

**Keywords:** beet molasses, salt stress, antioxidants, carbohydrates, proteins, amino acids, wild thyme

## Abstract

The growing demand for *Thymus serpyllum* biomass to produce drugs, cosmetics and spices necessitates the search for innovative methods mitigating the negative effects of environmental stressors in order to improve its yield under unfavorable conditions. Due to the exposure of plants to salinity stress (SS), we investigated the effect of sugar beet molasses (SBM) on the growth and biochemical parameters related to plants’ response to SS. Wild thyme plants were treated for 5 weeks to sodium chloride and 3% molasses solution using two modes of application (soil irrigation or foliar sprays). Plants irrigated by SBM showed slighter stem growth inhibition than control plants, high stress tolerance index and maintained a constant root water content under salt stress. Moreover plants treated with 100 mM NaCl and soil-applied SBM had lower lipid peroxidation level, showed lower POD activity, higher total soluble protein content and maintained a more even free amino acids level, compared to the control treatments. The concentration of potassium ions was higher in the case of plant roots irrigation with sugar beet molasses compared to control plants. In this experiment, most of the growth and biochemical parameters from foliar molasses-sprayed plants did not differ significantly from the control. We provided evidence that soil-applied SBM beneficially changed the plant’s biochemical response to salt stress. On the basis of the obtained results, we conclude that this soil amendment contributes to the strengthening of plant protection against this harmful environmental factor.

## 1. Introduction

The genus Thymus includes numerous species of economic importance [1,2]. Thyme was ascertained an important source of medicinal substances, with anti-allergic and anticancer (antitumor), i.e., cytotoxic properties [3,4]. The shrubby perennial wild thyme (*Thymus serpyllum* L.), native to Europe, has been usually applied to treat respiratory and gastrointestinal problems [5]. Recently, different secondary metabolites of wild thyme have become popular as important plant-derived products, with a growing number of applications due to their antimicrobial, antioxidant and essential oil properties [4,5,6,7]. Hence, the use of this wild plant in the food, pharmaceutical and cosmetic industries is increasing [5,8]. Therefore, we currently see an increased interest in researching the therapeutic properties of this species which serves as a high quality source of material with a wide variety effects [9]. Growing consumer interest in natural products, rather than synthetic chemicals, has further intensified research into the medicinal and aromatic plants, with *T. serpyllum* occupying an important place.

A huge contemporary problem concerns soil salinity, perceived as one of the main abiotic stresses, which significantly limits crop production. High soil-salt concentrations due to natural processes or disturbances resulting from irrigated agriculture inhibit the growth of plants and have a clearly negative effect on yield. Reduction in yields of some important crops can be as high as 50% [10,11]. This type of stressor causes osmotic destabilization in the root resulting in oxidative stress by excessive ROS production, and it also generates nutritional imbalances and ion toxicity [12,13]. Due to the damaging effect on crops, much attention has been paid to the development of effective strategies allowing to reduce the deleterious effects of salt stress on cultivated species [14]. Increasing stress tolerance in plants has clearly positive consequences in agriculture and horticultural production [15,16]. However, this is not an easy task as salt tolerance is a complex trait that is controlled by multiple genes and involves various biochemical and physiological mechanisms [17]. Mechanisms that enhance the degree of plant tolerance may also be induced or enhanced by the application of some chemicals [18,19,20]. Many strategies, including the use of certain organic compounds, such as amino acids, glycine-betaine, salicylic acids or plant growth promoting fungi/bacteria, mitigate salt stress [21]. The application of soil amendments, such as sugar beet molasses, to alleviate stress will allow crops to be grown in marginal areas that are currently unavailable. However, before their practical use, it is necessary to know exactly how these agents affect the key physiological processes that may determine plant productivity.

Molasses is the main by-product of sugar production. Sugar beet molasses is produced annually in large amounts. This by-product is used in animal feeding [22] and ethanol production [23]. It is characterized by a brown color and contains some poorly biodegradable derivatives of phenolic compounds such as melanin and melanoidins. Thus, acidic molasses wastewater effluent results in the environmental pollution of water bodies. There are also reports that such waste can serve for soil microbiota as a source of carbon, minerals, vitamins and antioxidants [24,25]. The agricultural use of sugar beet molasses has been shown to stimulate nutrient uptake and biological activity of arable soil [26]. The nutrients from beet molasses may be therefore useful for crop production and could partially replace some of the chemical fertilizers required by crops. It was found that the use of molasses had a positive effect on the physical structure of the soil and increased the biological activity of beneficial microorganisms [27,28,29]. Molasses contains a fairly high concentration of organic carbon and mineral elements, including nitrogen, phosphorus, potassium, magnesium, calcium, sulfur, iron, some important micronutrients, vitamin B and biotin [23,30,31].

We hypothesized that ingredients present in sugar beet molasses increase the level of *T. serpyllum* tolerance to salinity stress. We applied this by-product from the sugar production in two different forms, that is soil irrigation and foliar fertilization (in fact, in a case of the studied plant, it was shoot irrigation). To the best of our knowledge, the interactions between different forms of molasses addition to alleviate the response of aromatic plants with medicinal properties, such as wild thyme, to salt stress have not yet been verified. A careful investigation of these relationships may enable the wider use of this plant of economic importance in marginal areas.

## 2. Results

### 2.1. Soil pH and EC Analysis

The maximum pH was found in 200 mM sodium chloride-treated soil (6.44) and the lowest pH level was found in non-salt-treated soil (5.54), as shown in Table 1. Electro-conductivity (EC) values in soil samples where NaCl was not applied (0 mM) was 0.98 dS m^−1^ at the end of the experiment. When salt stress was applied, EC increased in parallel to the sodium chloride concentration, and was, respectively, 3.22 (moderately salinized soil—100 mM) and 4.64 dS m^−1^ (strongly salinized soil—200 mM). The addition of beet molasses for both soil and foliar application did not change either pH or EC during whole experimental scheme.

### 2.2. Growth Parameters

**Figure 1:**
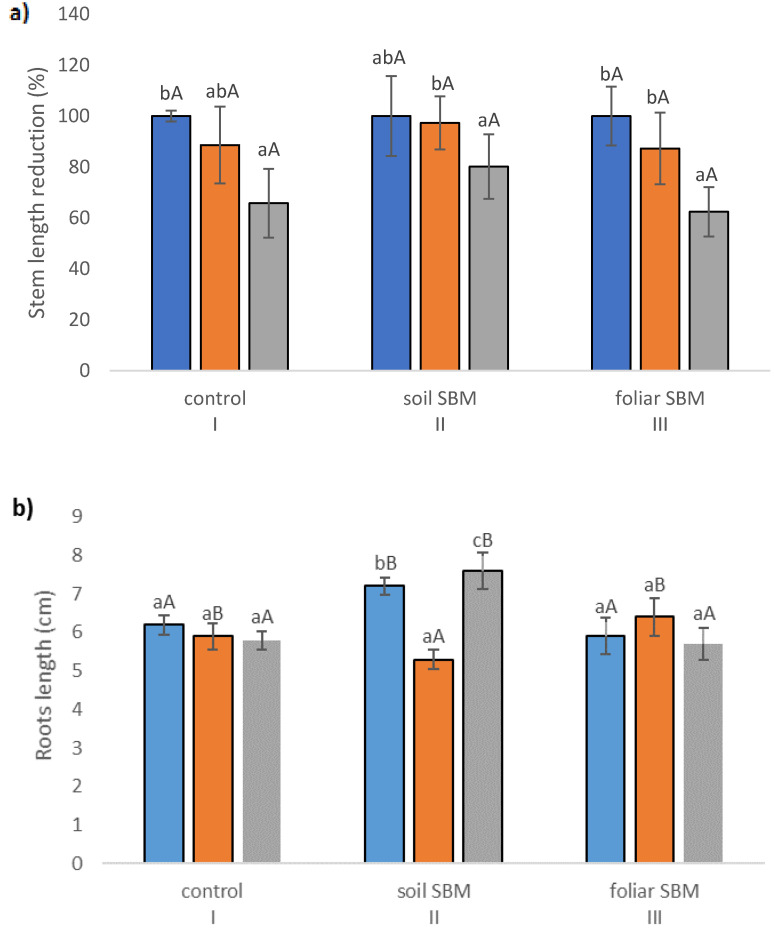

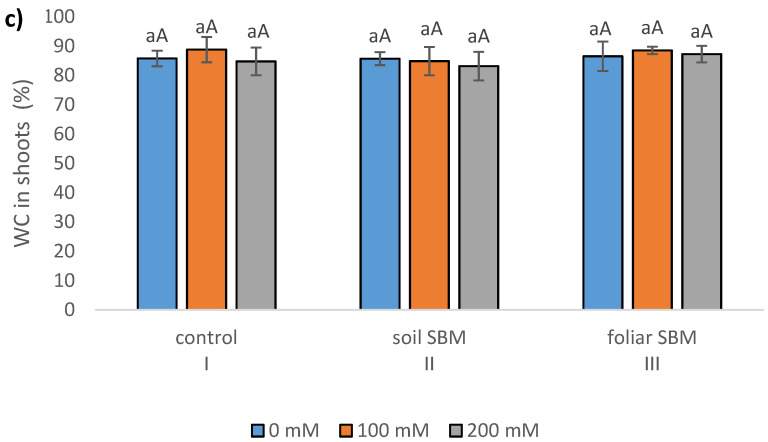

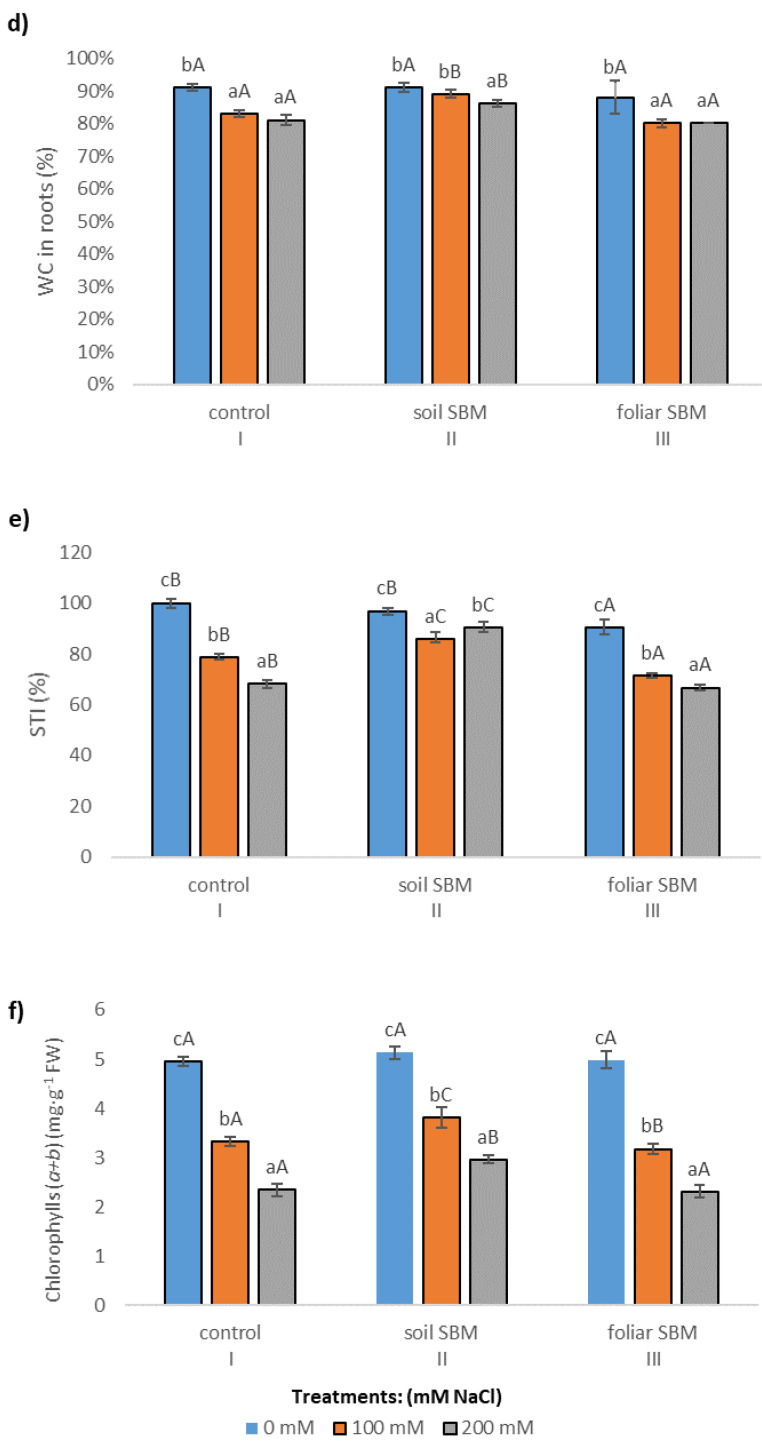
Stem length reduction (%) (**a**); roots length—(**b**); water content in leaves (%)—(**c**); water content in roots (%)—(**d**); stress tolerance index (STI)—(**e**); total chlorophylls (Chl *a + b*) (**f**) in the *Thymus serpyllum* plants after five weeks of salt stress and sugar beet molasses treatments (soil SBM—sugar beet molasses applied to the soil; foliar SBM—shoot application of sugar beet molasses). Stem length is shown as percentages of the corresponding non-stressed controls, taken in each case as 100%. Values shown are means ± SD (n = 7). For each variant of the experiment, lowercase letters indicate significant differences between NaCl level and different capital letters indicate significant differences between each variant experiment for the same NaCl level, according to Tukey’s test (α = 0.05).

The stem length (SL), expressed as a percentage of respective untreated controls, was lowest in *T. serpyllum* exposed to 200 mM NaCl in all three experimental variants, that is with two different SBM mode application and control without its application. However, the reduction was slight and statistically insignificant under application of SBM solution to the soil. Stem length proved to be shorter by about 35, 20 and 38% under 200 mM SS with comparison to 0 mM SS in variants I, II and III, respectively (Figure 1a). Regarding root length, the results were unambiguous: the shortest roots (5.35 cm) were found under 100 mM sodium chloride in variant II, while the longest under 200 mM NaCl in the same variant (7.62 cm); whereas the length of the roots in non-salt-treated plants was on average 7.24 cm. However, these values differed significantly compared to the values obtained in the first and third experimental variants with the same sodium chloride level. For variant I (without SBM) and III (with foliar SBM application), no significant differences in root length were found under the same sodium chloride level, as well as between different levels of NaCl in the case of the same method of SBM application (Figure 1b). Water content percentage in the shoots did not change significantly with both sodium chloride applications with SBM, and ranged from 84 to 88% (Figure 1c). On the other hand, WC% reduction in the roots was more pronounced under salt stress conditions (200 mM) in variant I (81%) and variant III (80%), in comparison to variant II with the value 86% (Figure 1d). The stress tolerance index (STI) for shoots exposed to 0, 100 and 200 mM sodium chloride amounted to 100, 79, 68, respectively, with the values referring to the variant I. Whereas, in the variant II, the differences with STI for each NaCl level were not substantial and amounted to 97, 86 and 90%, respectively. While in the case of the variant with foliar application of SBM, the stress tolerance index reached 90, 71 and 67 for 0, 100 and 200 mM treatment NaCl, respectively (Figure 1e).

### 2.3. Biochemical Parameters

#### 2.3.1. The Chlorophyll Content

The content of total chlorophyll (*a + b*) was taken as a parameter reflecting the plant’s ability to photosynthesize and indirectly the ability to support its growth under stressful conditions. Salinity reduced the total chlorophyll (*a + b*) content in the shoots in all three variants of the experiment. A pronounced decrease was observed in variant I and III of the experiment and a slighter decrease was visible in variant II. The decline reached 33%, 26%, 36% under 100 mM NaCl, 53%, 42%, 54% under 200 mM NaCl in comparison to 0 mM NaCl, respectively, in variant I, II and III of the experiment (Figure 1f).

#### 2.3.2. MDA and Membrane Stability

**Figure 2:**
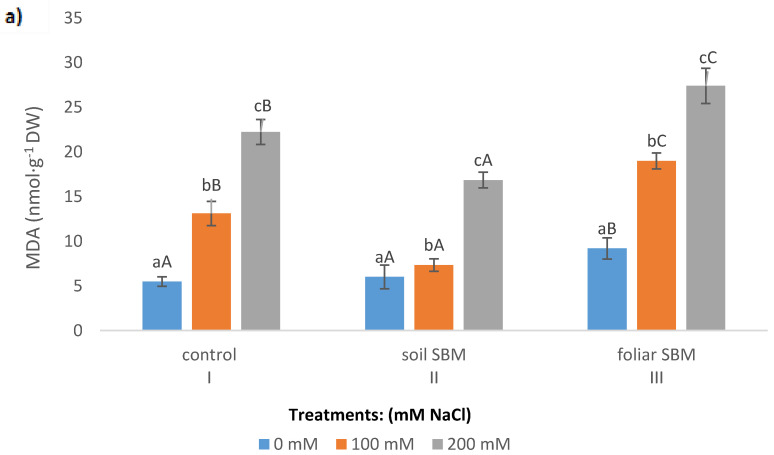

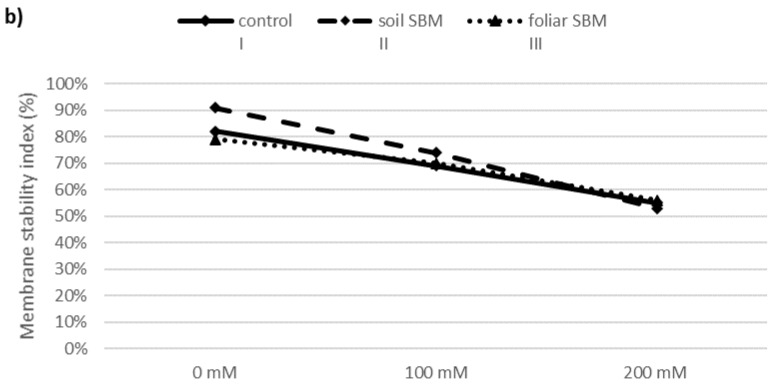
Malondialdehyde (MDA) (**a**) and membrane stability index (MSI) (**b**) in the shoots of the *Thymus serpyllum* plants after five weeks of salt stress and sugar beet molasses treatments (soil SBM—sugar beet molasses applied to the soil; foliar SBM—shoot application of sugar beet molasses). Values shown are means ± SD (n = 7). For each variant of the experiment, lowercase letters indicate significant differences between NaCl level and different capital letters indicate significant differences between each variant experiment for the same NaCl level, according to Tukey’s test (α = 0.05).

Lipid peroxidation expressed as a malondialdehyde (MDA) content, showed increasing trend over the sodium chloride concentration in all tested variants of the experiment. SBM application to the soil maintained the same MDA level under 100 mM as under 0 mM NaCl. However, this alleviating effect was not observed under 200 mM NaCl and MDA amount increased significantly in all three variants of the experiment and reached 22.24, 16.85 and 27.38 µmol·g^−1^ FW in I, II and III variants, respectively (Figure 2a). The membrane stability index in shoots of *T. serpyllum* under salt stress showed a decreasing trend with NaCl concentration. Under non salt stress condition MSI was slightly higher (about 10%) in II variant of the experiment compared to variant I (control) and variant III (foliar-applied SBM) (Figure 2b).

#### 2.3.3. Antioxidants

**Figure 3:**
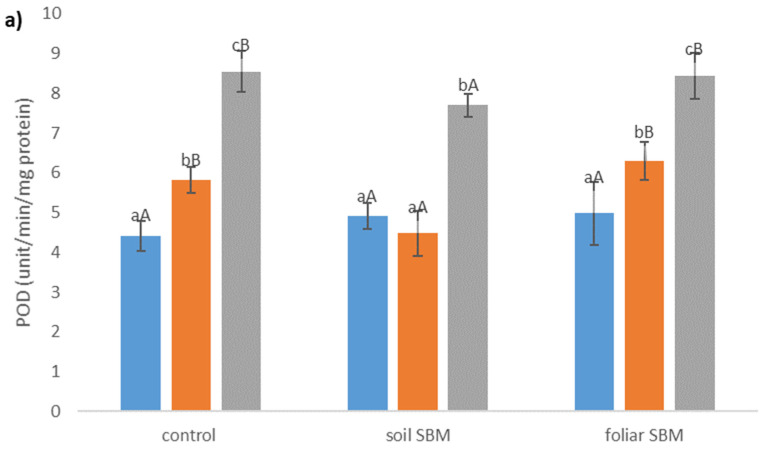

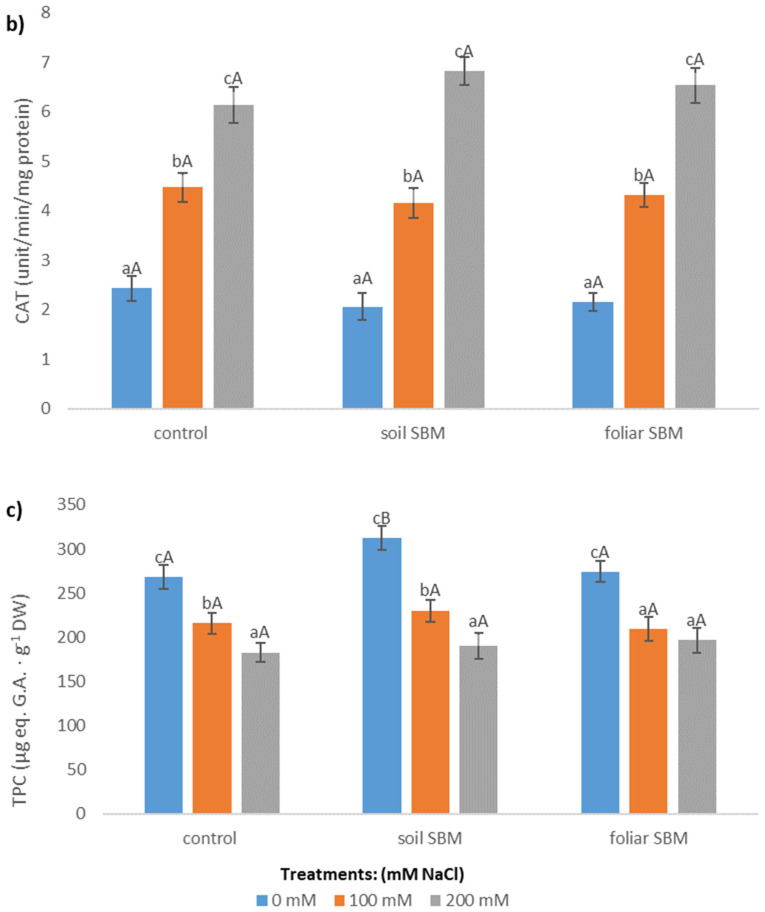
Peroxidase activity (POD) (**a**); catalase activity (CAT) (**b**) and total phenolic compounds content (TPC) (**c**) in the shoots of the *Thymus serpyllum* plants after five weeks of salt stress and sugar beet molasses treatments (soil SBM—sugar beet molasses applied to the soil; foliar SBM—shoot application of sugar beet molasses). Values shown are means ± SD (n = 7). For each variant of the experiment, lowercase letters indicate significant differences between NaCl level and different capital letters indicate significant differences between each variant experiment for the same NaCl level, according to Tukey’s test (α = 0.05).

The POD and CAT activity were enhanced in plants grown under salt stress conditions. POD activity was lower in soil SBM treated plants (about 23% and 10% under 100 and 200 mM NaCl, respectively) compared to control plants (variant I) while there were no differences in POD activity between control and foliar-SBM treated plants under the same NaCl conditions (Figure 3a). Regarding CAT activity there were not observed significant difference between all tested variants experiment under the same salinity level and CAT activities amounted to an average 2.22, 4.32, 6.5 under 0, 100 and 200 mM NaCl, respectively (Figure 3b). Total phenolic compounds shown decrement changes in the amount with increasing salinity level. Both tested method of SBM application to the salt-treated plants did not change the level of TPC under SS. In variant II of the experiment, the content of TPC under non salt stress condition was the highest and reached 312.56 µg eq. G.A g^−1^ DW (Figure 3c).

#### 2.3.4. Stress-Related Metabolites

##### Total Soluble Carbohydrates (TSC)

**Figure 4:**
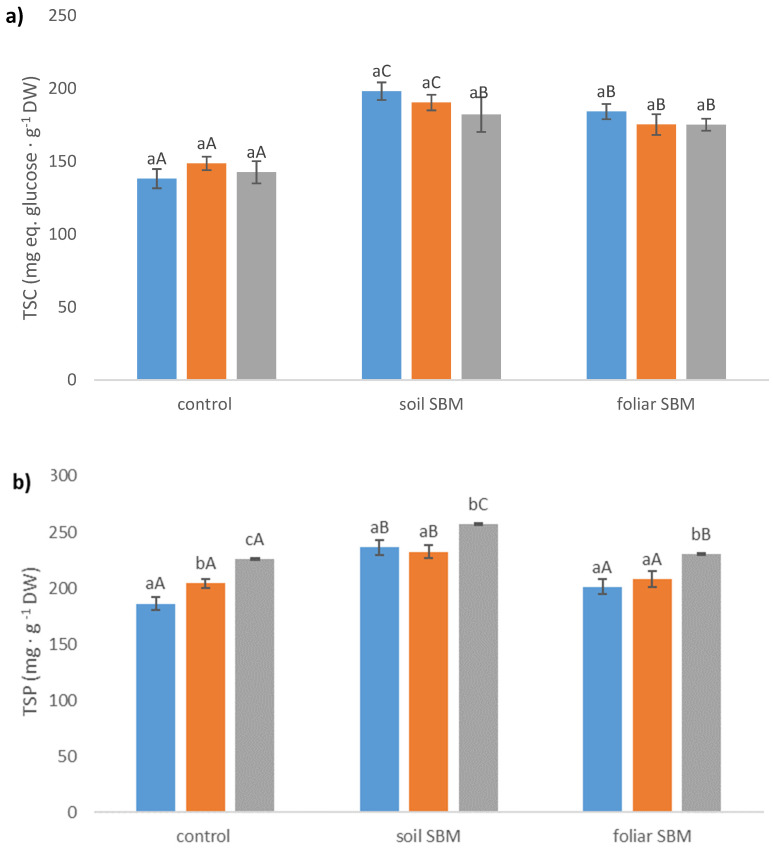

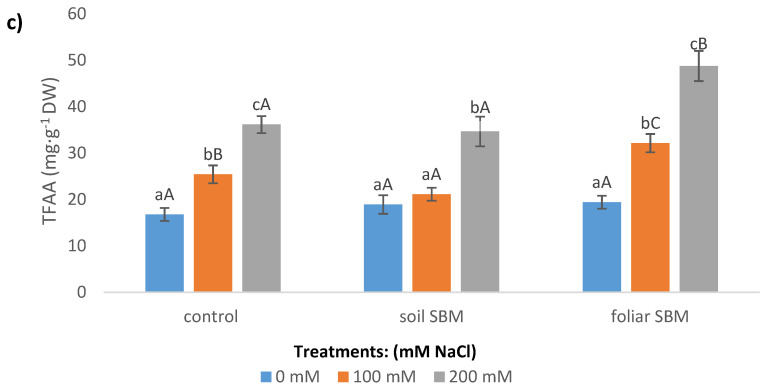
Total soluble carbohydrates (TSC) (**a**); total soluble proteins (TSP) (**b**); total free amino acids (TFAA) (**c**) content in the shoots of the *Thymus serpyllum* plants after five weeks of salt stress and sugar beet molasses treatments (soil SBM—sugar beet molasses applied to the soil; foliar SBM—shoot application of sugar beet molasses). Values shown are means ± SD (n = 7). For each variant of the experiment, lowercase letters indicate significant differences between NaCl level and different capital letters indicate significant differences between each variant experiment for the same NaCl level, according to Tukey’s test (α = 0.05).

Total soluble carbohydrates content did not change significantly in all three variants of the experiment under applied salt stress; however, much higher values of this compound were recorded in variants II and III under the same SS level with comparison to variant I. The increment of TSC was about 30%, 19%, 16% for 0, 100 and 200 mM NaCl, respectively, under soil SBM application and (about 25%, 14%, 19% for 0, 100 and 200 mM NaCl, respectively, under foliar SBM application compared to variant I of the experiment. The highest content of total sugars was recorded when sugar beet molasses was applied to the soil (II) and reached 198.22 mg eq. glucose g^−1^ DW under non salt stress condition (Figure 4a).

##### Total Soluble Protein (TSP)

Total soluble protein content was higher in variant II of the experiment under both non salt stress and salt stress conditions compared with variant I and III. It should be pointed out that TSP increment corresponded with the increase in salt concentration in all three variants of the experiment, except variant I and 100 mM NaCl conditions where TSP amount did not increase in comparison to 0 mM NaCl which has been observed the remaining two of them. The highest TSP was recorded in variant II under 200 mM and reached 257.22 mg·g^−1^ FW (Figure 4b).

##### Total Free Amino Acids (TFAA)

In the presence of the salinity the TFAA content increased significantly in variant I and III of the experiment, whereas in variant II of the experiment the increase was observed only under higher sodium chloride level i.e., 200 mM. The addition of SBM in both application modes did not change the TFAA amount under non salt stress condition in comparison to the control (variant I), however, foliar SBM application has resulted in higher levels of those compounds under 100 and 200 mM compared to variant I and II under the same SS level (Figure 4c).

#### 2.3.5. Ions

**Figure 5:**
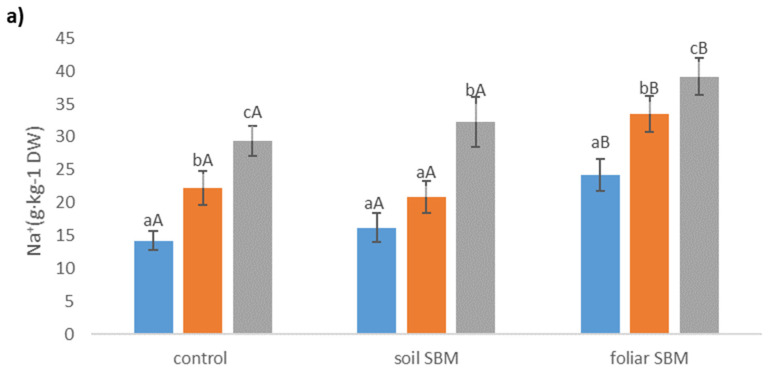

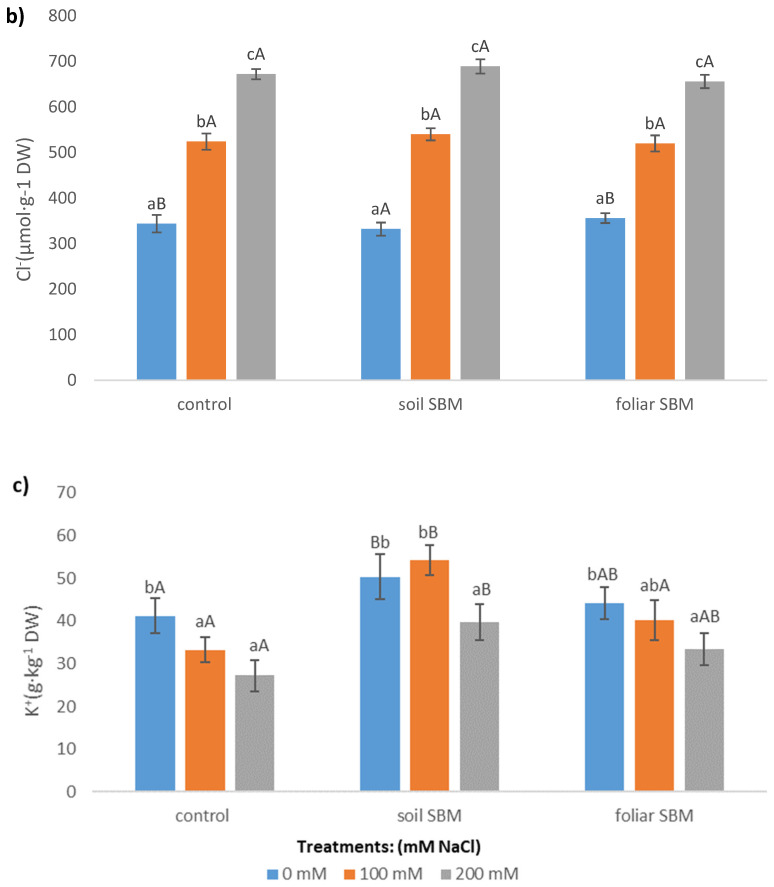
The sodium (Na^+^) (**a**), chloride (Cl^−^) (**b**), potassium (K^+^) (**c**) concentrations in the shoots of the *Thymus serpyllum* plants after five weeks of salt stress and sugar beet molasses treatments (soil SBM—sugar beet molasses applied to the soil; foliar SBM—shoot application of sugar beet molasses). Values shown are means ± SD (n = 7). For each variant of the experiment, lowercase letters indicate significant differences between NaCl level and different capital letters indicate significant differences between each variant experiment for the same NaCl level, according to Tukey’s test (α = 0.05).

Sodium and chloride levels increased in the shoots of *Thymus serpyllum*, in parallel with increasing external salinity, regardless of the SBM application. Plants treated by foliar SBM accumulated more Na^+^ in their upper-grounds organs than plants from variants I and II under the same SS level (Figure 5a). Regarding chloride ions, there were no recorded differences between the three variants of the experiment for the same SS level (Figure 5b). Potassium content decreased under SS, however, in variants II and III of the experiment, decrement was observed only under higher NaCl level (200 mM). The concentration of this ion was highest when SBM was applied to the soil (Figure 5c).

#### 2.3.6. Data Correlations

##### Correlations Matrix

**Figure 6:**
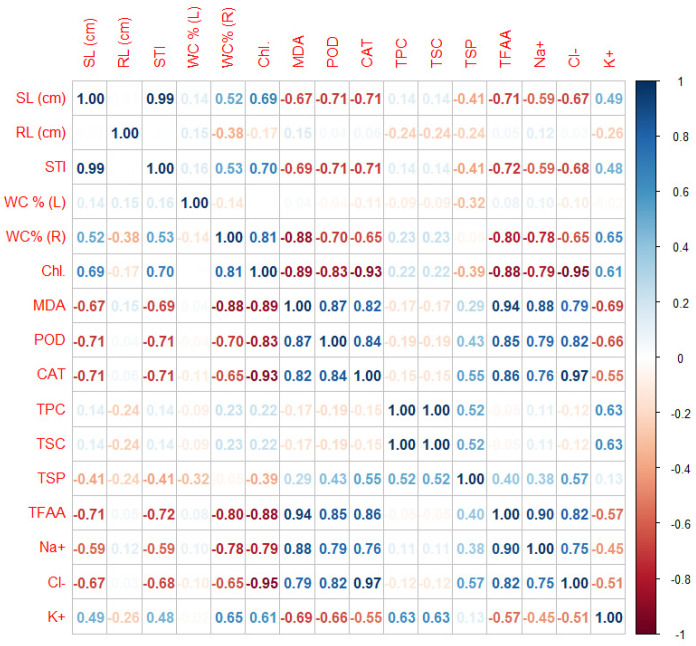
Pearson’s linear correlation for all measured biochemical parameters of *Thymus serpyllum* plants after five weeks of salt stress and sugar beet molasses treatments.

STI and SL (cm) were most correlated with each other. A high correlation was found for CAT and Cl- and between MDA and TFFA. High correlations were also observed for the TFFA and Na+ parameters (Figure 6).

**Figure 7:**
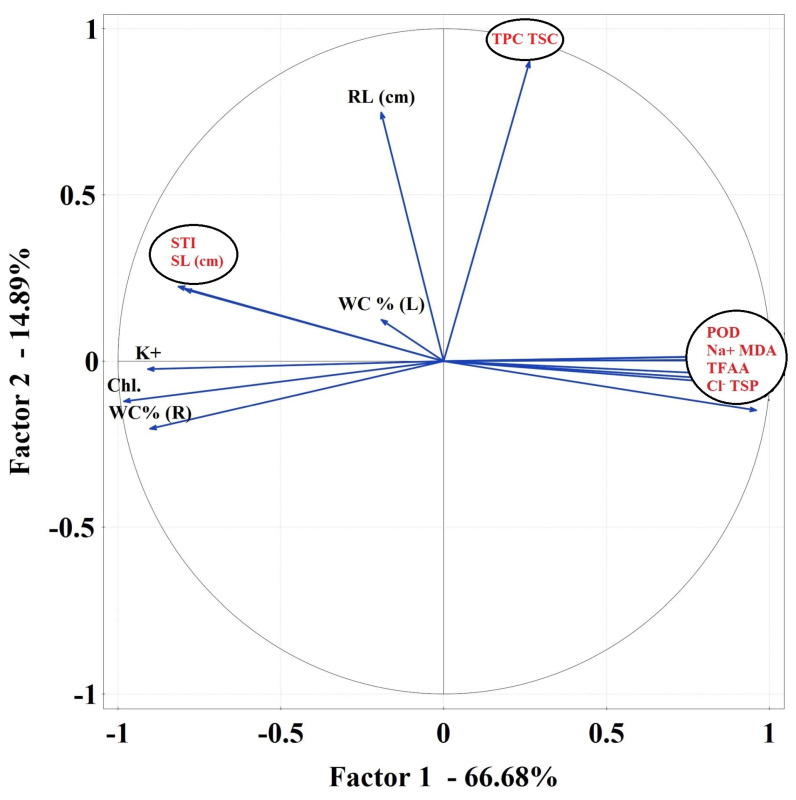

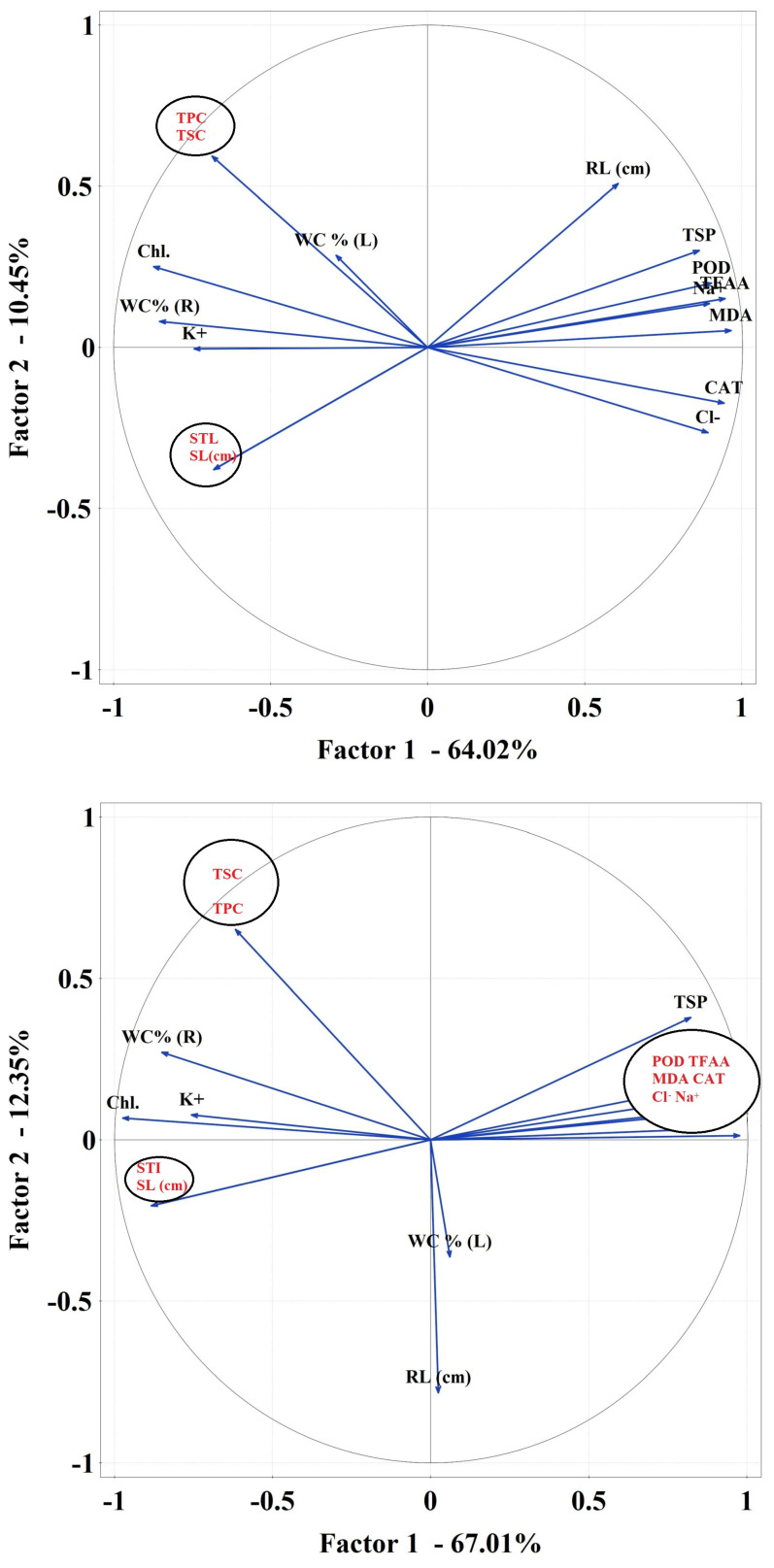
Principal component analysis (PCA) using plot showing all biochemical parameters separately for the first variant of an experiment—salt stress without sugar beet molasses application (**a**), for the second variant of the experiment—salt stress with soil sugar beet molasses application (**b**) and third variant of the experiment—salt stress with foliar sugar beet molasses application (**c**).

PCA 1 analysis showed strong relationships between the studied parameters. WC (%) R formed a negative bond with Chl (Figure 7a). In PCA 2 analysis, RL, TSP and POD, Na^+^, MDA were very strongly related. Linear combinations of explanatory variables were revealed (Figure 7b). A strong association has been noted for TSP, POD, TFAA, Na^+^, CAT, Cl^−^ and MDA in PCA 3 (Figure 7c).

**Figure 8:**
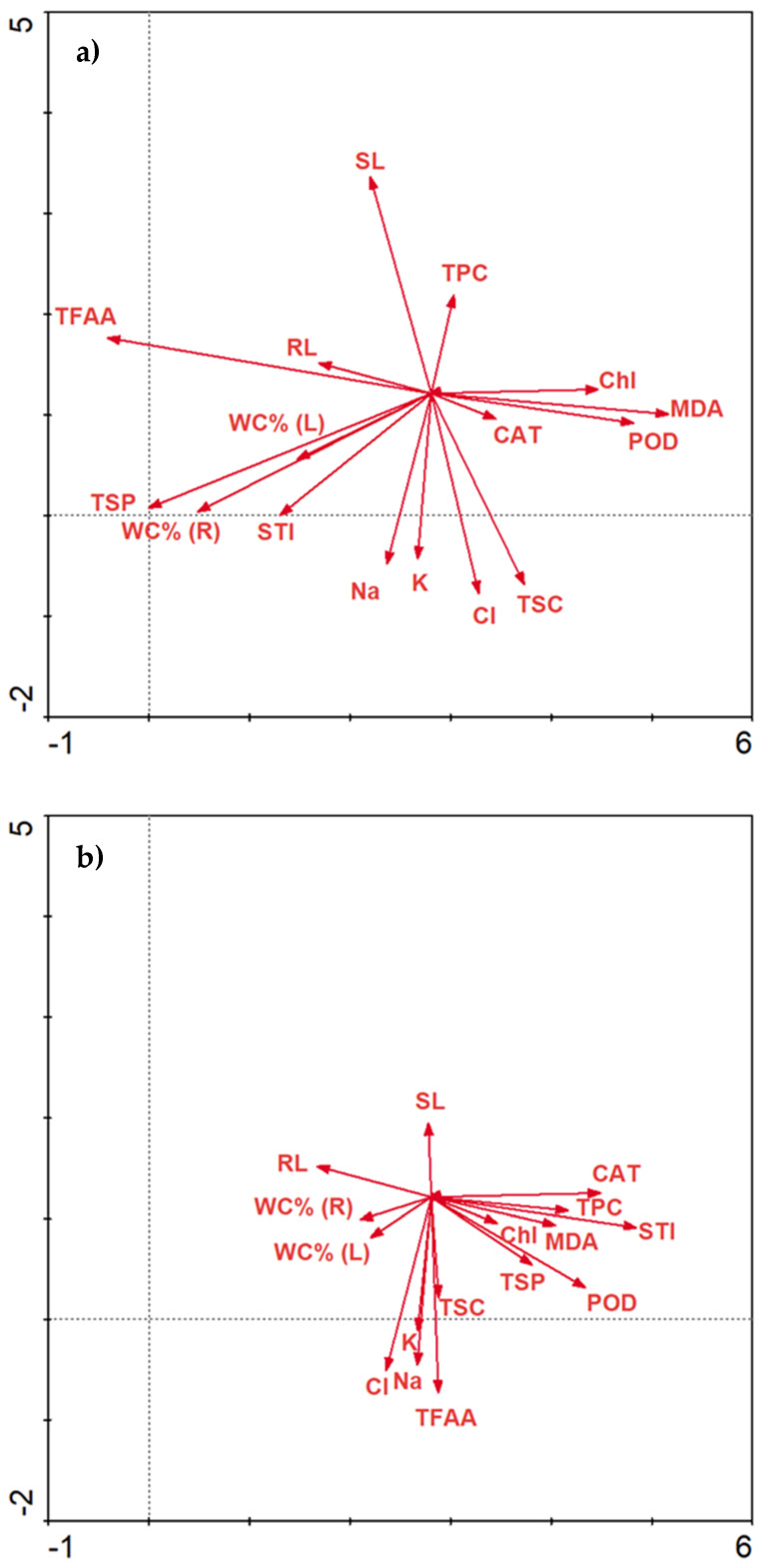

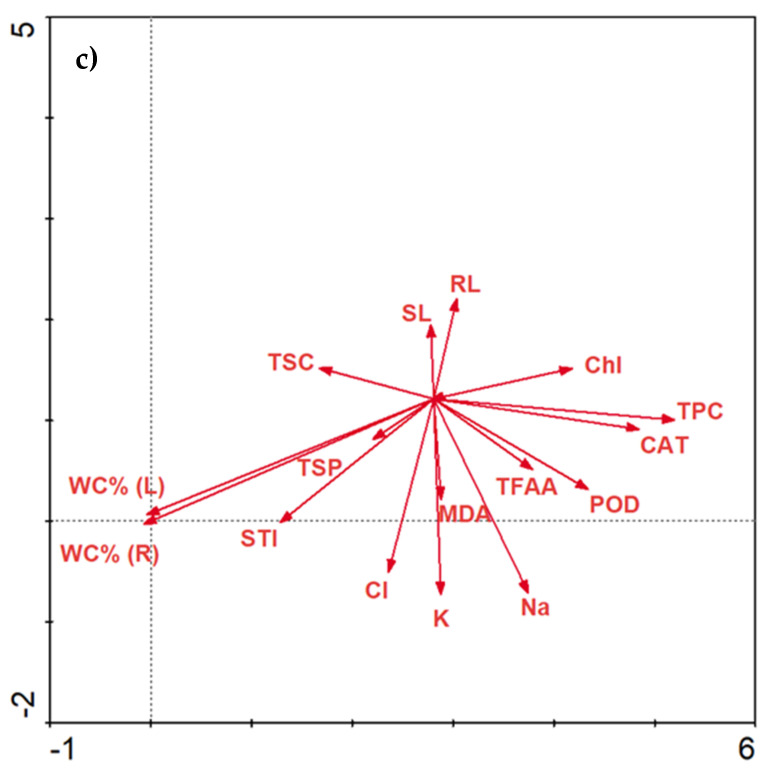
Redundancy analysis (RDA) of the data explained by environmental variables and treatments between salinity stress, molasses application and biochemical parameters. RDA 1—salinity stress at 0 mM NaCl level (**a**); RDA2—salinity stress at 100 mM NaCl level (**b**); RDA 3—salinity stress at 200 mM NaCl level (**c**).

The concentration of NaCl changes the activity of biochemical parameters. As NaCl increases, Chl, TPC and CAT are positively correlated with each other (Figure 8a). Increasing the concentration of NaCl may result in greater activity of MDA and STI (Figure 8b). Moreover, a higher increase in NaCl concentration affects the presence of TFAA (Figure 8c).

## 3. Discussion

Salinity adversely affects all growth aspects of glycophytic plants [32]. In many regions of the world the increased salt tolerance level of numerous species from *Lamiaceaeae* family is needed in order to maintain high yield [33]. This is especially true where the area of salt-affected soils are expanding due to constantly increasing temperatures and prolonged drought periods which cause increased soil salinity [12,34,35]. Therefore, in the present study, we examined whether the adverse effects of salt stress in *Thymus serpyllum* cultivation could be mitigated by the exogenous application of sugar beet molasses as a soil additive or in the form of plant spraying, and to what extend the application mode impacts the antioxidant plant system. In addition, we checked the levels of some metabolites in the wild thyme, which are often involved in the plant response to salinity stress. In a study of Bistgani et al. [36] on *Thymus vulgaris* and *T. daenensis*, based on metabolites profiling and antioxidant activity of herb, those species have been described as moderately tolerant to salinity. In our experiment *T. serpyllum* was also found to be a relatively high salt-tolerant plant because it was able to grow in the presence of 200 mM NaCl without obvious signs of injury. For this reason, our research concerning treatments that enable an increase in the tolerance to salinity stress for this selected European ecotype of *T. serpyllum* seems to be justified as then *T. serpyllum* could also be grown on strongly salinized soils.

There is a growing interest in the use of organic soil amendment as a smart alternative to over-fertilizing with chemicals. In horticulture, in the case of nutrients deficiency, foliar application of microelements to aromatic plants is used [37,38] and sometimes organic bio-stimulants are additionally applied with quite good results [39,40]. There are some reports on the use of molasses with intention to improve poorly structured or sandy soils [41]. Regarding the impact of SBM application methods, our data indicate that SBM spraying did not have a large impact on most of the measured plant biochemical parameters, as assessed by comparison with the control plant response. In that respect, soil addition with SBM gives good effects in the case of rapeseed [29] and okra plants (*Abelmoschus esculentus*) [42]. Additionally, regarding the example of *Lycopersicon esculentum* cultivation it was proved that the use of molasses resulted in the suppressed activity of the nematodes which is important for ensuring the good health of cultivated plants [43].

There are just a few reports indicating that organic compounds are able to minimize the adverse effects of stresses on *Thymus* plants. Najafian et al. [21] provided evidence that salicylic acid (SA) can effectively induce tolerance of *Thymus vulgaris* to salt stress. In their research, application of SA increased water use efficiency, rate of photosynthesis and thus mesophyll efficiency in salt stressed *T. vulgaris* plants. Moreover, the beneficial effects of SA in saline conditions include the sustained transpiration activity and consequently enable reasonable biomass growth, and had contributed to necrosis avoidance or the reduction of accelerated leaf aging, depending on stress severity. By contrast, Khalid’s research group [44] investigated to what extent magnesium ions impact vegetative growth and several biochemical compounds in *Thymus*. The Mg2^+^ treatment resulted in a significant rise of parameters characterizing the vegetative growth of thyme, the plant photosynthesis pigments, total sugars, protein and some nutrient contents. In our study we demonstrated that short-term soil treatment with sugar beet molasses modified some metabolic features which led to inducing tolerance in wild thyme to mild salinity stress. The molasses is rich in organic carbon, vitamins and different nutrients [45]. The content of organic matter is the important parameter determining soil fertility and allows to increase the efficiency of nutrients by increasing water holding capacity and nutrient retention. This ensures the nutrients are taken up by the plant roots for a long time [46]. This agrees with the findings of others that reported that molasses induce tolerance to many biotic and abiotic stresses. Hatano and Yamatsu [47] applied SBM meladoinoidin-like products to obtain tolerance to elevated lead (Pb) levels in soil, and by such treatment they enhanced the Pb phytoextraction of three plant species representatives of the *Brassicaeae* family, rich in wild species, that has already shown characteristics of phytoaccumulating heavy metals [48]. The organic biomass-rich amendments were also applied with the aim of soil-born pathogen and nematode control [49]. In our study, plants irrigated by SBM to the soil showed slight stem growth inhibition, high stress tolerance index and maintained a constant root water content in comparison to untreated plants, even during NaCl treatment. Higher STI is related to enhanced biomass production which comes from the plants’ nutrient supply by molasses. An increase in plant biomass, root vigor and root development under condensed molasses soluble was also reported by Li et al. [29], however, a higher concentration of molasses solution which inhibited plant rapeseed growth was used in their experiment. Chemical composition and percentage of water content in molasses varies depending on the origin of this by-product. *Thymus serpyllum* is a wild-growing plant in contrast to the rapeseed cultivar, ‘Zhoengshuang’, described by Li et al. [29]. Most of the wild plants from the Lamiaceae family are usually found in a wide variety of habitats and tend to be more tolerant of the trophic conditions of the habitats in which they occur. These are plants from a wide ecological scale. For this reason, higher doses of SBM did not affect negatively examined parameters of *Thymus serpyllum*. Moreover, SBM could act as a soil amendment improving the soil’s physical and chemical properties. The enhanced biomass production can also be due to the high sucrose content in molasses which helps to maintain osmotic balance and turgor pressure, ensuring a prolonged water and nutrients uptake under stress (both necessary for photosynthesis) [50,51]. By comparison, higher levels of chlorophylls in SBM-treated *T. serpyllum* plants may be connected with the increased supply of magnesium which is present in the applied molasses. Magnesium is a constituent of the chlorophyll molecule and is required for the normal structural development of the chloroplast [52]. Regarding the potential role of molasses in the antioxidant properties of plants, our results showed lower level of lipid peroxidation and less peroxidase (POD) activity in salt and soil SBM-treated plants. The relative activity of POD in SBM-treated plants under salt stress conditions observed in this study may be related to the ability of molasses to scavenge free radicals, thus reducing the activation of the antioxidant machinery in *T. serpyllum* plants under salt stress. The exogenous application of sucrose alleviates the oxidative stress caused by osmotic imbalance, and the consequent ROS buildup, by inducing antioxidant defense mechanisms [53,54]. There is also evidence that applied exogenous sugars feed into different defense pathways such as proline accumulation, crucial for cytoplasmic osmotic balance adjustment, ROS scavenging, etc. [55]. The application of molasses increased the rate of development of sugarcane seedlings by regulating the antioxidant enzyme systems to reduce ROS damages [56]. On the other hand our results are in contrast to studies by Li’s research group [29] where molasses solutions increased the activity of superoxide dismutase, peroxidase and catalase. We assume that in the experimental setup described here a 3% solution of sugar beet molasses syrup containing 20% water can efficiently reduce oxidative stress by scavenging active oxygen species arising during stress. In our study, sugar beet molasses applied to the soil induced growth in total soluble protein content in plants under all sodium chloride levels. The increased content of soluble protein content in plants and thus the reduction of adverse effects of the presence of salt in the soil can be attributed to a certain pool of amino acids in the molasses which improve the plant’s nutritional status and its response to salt stress [57,58]. In our study, sugar beet molasses could enrich the studied plants with proteins necessary to initiate plant tolerance to osmotic and oxidative stresses caused by high salt concentration. Several functional groups of proteins are involved in signaling, changes in gene expression, protein biosynthesis and degradation and the resulting changes in the relative abundance of proteins involved in plants’ response to salt stress [59,60]. Moreover, our results show the total free amino acids level was maintained both under control and NaCl treatments. Total free amino acids are important plant signaling molecules induced by stress conditions. Very often the content of TFAA in the above-ground organs increases under the influence of salt stress and this is a plant’s response to the abiotic stresses [58,61]. In addition to sugar, several other compounds are presently separated from beet molasses. One of these compounds is glycinebetaine (GB), an amino acid derivative accumulated in many plant species grown under stress. GB is assumed to have several adaptive effects on drought and salt stressed plants. The role of GB is to maintain water content in plant cells by lowering solute potential under osmotic stress [62,63].

In the present work we also observed higher concentrations of potassium ions when plants were treated by sugar beet molasses. It is known that molasses, in addition to calcium, magnesium or iron, is rich in potassium [31], which plays a crucial role in plants’ salt tolerance mechanisms. One significant effect of salt stress on K^+^ homeostasis is Na^+^—induced K^+^ efflux from both root and leaf cells [64,65]. This efflux has been established to be the result of excess Na^+^ influx into the cytoplasm, leading to the depolarization of the membrane potential below resting potential, with a consequential activation of K^+^ outward channels. Thus, the ability of plant cells to prevent membrane depolarization by maintaining a more negative inside potential, in order to enhance inhibition of K^+^ efflux, constitutes a stress tolerance mechanism [65,66]. The capacity to retain intracellular K^+^ is also crucial for salt stress tolerance [67]. Moreover potassium is required by a large number of enzymes involved in plants’ adaptation to different kinds of abiotic stresses [68]. Šarić et al. [69] reported that sugar beet molasses can be used as a hypertonic solution in the osmotic dehydration of plant material. The researchers showed it is an excellent medium for the osmotic dehydration of fruits and vegetables primarily due to a high content of dry matter (80%, *w/w*) and specific nutrient content. This feature of molasses can also be beneficial in plant protection against salt stress which causes osmotic stress. An important advantage of using sugar beet molasses as a hypertonic solution is an enrichment of the dehydrated plant material in minerals and vitamins, which are uptaken from molasses into the plant tissue. Here, the concentration of applied molasses can be significant because the uptake of vitamins by plant roots can be a linear function of vitamin concentration in the uptake solution [70].

It is worth noticing that molasses ingredients can sometimes serve as a signaling function, leading to the expression of plants tolerance. Therefore, this effect should be carefully investigated in the near future. Induction of multiple stress tolerance in plants through the use of SBM and its derivatives may have a significant practical application in agriculture, horticulture and forestry if research in this direction is carried out with appropriate intensity.

## 4. Materials and Methods

### 4.1. Plant Material and Experimental Design

*Thymus serpyllum* L. seed samples were obtained from Polan Sp. z o,o. The seeds were sown in trays containing a mixture of standard soil, peat and perlite (1:1:1), and were placed in a greenhouse with regulated temperatures of 23–17 °C (day/night), and 70% relative humidity. Eight weeks after sowing, plantlets were transferred to individual pots on the same type of substrate as for germination phase.

During the experiment we used the sugar beet molasses (SBM) composed of: 20% of water, and in the 80% of solid mass there were about: 60% of total sugars (sucrose, glucose, fructose, raffinose, galactose, etc.), 5.5% of amino-acids, 0.3% of citric acids, 1.9% of K, 0.1% of Na, 0.4% of Mg and other elements.

The experimental design consisted of three substrate salinity levels: 0, 100 and 200 mM NaCl, and following modes of sugar beet molasses (SBM) application (variant I—control: non SBM; II: soil SBM application by irrigation; III: foliar fertilization by spraying the SBM solution). Sugar beet molasses were applied in two ways—by irrigation (variant II) of 300 mL 3.0% molasses solution, and by foliar spraying (variant III of the experiment) of the same molasses volume twice per week (one day after sodium chloride application). The foliar spray was repeated twice a week until plants were harvested, with a total of 10 sprays. To each treatment, 83.7 g of SBM was added throughout the whole experiment. Considering the fact that SBM contains 20% of water it produces 67.0 g of condensed beet molasses. Control was water-irrigated with the same amount and frequency as other treatments. Salinity levels were adjusted by the addition of sodium chloride solution to the substrate. Electrical conductivity (EC) was measured before NaCl application and after 5 weeks of the respective treatment. Irrigation by NaCl solution was carried out twice a week by adding 300 mL of salt solution to each pot (16 cm in diameter). The experiment was laid out in a randomized complete-block design with 7 replicates per treatment. Each replicate was composed of two plants (two plants per pot) resulting in a total of 126 plants. After 5 weeks, all the plants were estimated for growth and harvested for biochemical analyses. Fresh plant material was stored frozen at −80 °C, and dry material in tightly closed containers at room temperature.

### 4.2. Soil pH and EC Analysis

Soil pH and electrical conductivity (EC) was measured after five weeks of experimental treatments. Soil samples taken from pots of the same treatment, were air-dried and then passed through a 2-mm sieve. A mixture of soil:water (1:1 for pH and 1:5 for EC) suspension was prepared using deionized water at 20 °C and mixed for one hour at 600 u/min. pH was determined using an electrode potentiometrically by Crison pH-Meter Basic 20 (Crison, Barcelona, Spain), and electric conductivity was determined using a Crison Conductivity Meter 522 (Crison, Barcelona, Spain) and expressed in dS m^−1^.

### 4.3. Assessing Plant Growth

At the end of the experiment the plant material was collected and the following growth parameters were measured: stem length (SL), root length (RL), fresh weight (FW), water content percentage (WC%) of above-ground parts and roots separately. FW was measured by weighing the total mass of the shoots and roots after harvesting. A fraction of the fresh material was dried at 65 °C until constant weight, to obtain the dry weight (DW), which was used to calculate the shoots and roots water content, in percentage, for each plant:

WC = ((FW − DW)/FW) × 100

Stress tolerance index (STI) was calculated for shoot dry biomass using the following formula:

STI = (mean DW of salt stress-treated shoots/mean DW of non salt stress-treated shoots) × 100

### 4.4. Assessing Plant Biochemical Parameters

Chlorophylls content. After extraction in 80% acetone at 4 °C (100 mg tissue in 10 mL of acetone) samples were centrifuged for 15 min at 4800× *g*. Then, the supernatant was filled up to 10 mL volume. Chlorophyll a (Chla) and chlorophyll b (Chlb) were determined by measuring absorbance of the extract at 470, 646 and 663 nm using a double beam spectrophotometer U-2900 (Hitachi High-Technologies Corporation, Tokyo, Japan). Next, the pigment content was calculated in accordance with the equations presented by Lichtenthaler and Buschmann [71].

Malondialdehyde (MDA). MDA, a final product of membrane lipid peroxidation, a reliable marker of oxidative stress, was determined as described by Hodges et al. [72]. Extracts were mixed with 0.5% thiobarbituric acid (TBA), prepared in 20% trichloroacetic acid (TCA) (or with 20% TCA without TBA for the controls), and were then incubated at 95 °C for 20 min. After stopping the reaction on ice, the supernatant’s absorbance was measured at wavelength 532 nm. The non-specific absorbance at 600 and 450 nm was subtracted and the MDA concentration was calculated with the following equation:

MDA = 6.45(D532 − D600) − 0.56D450

Membrane stability index (MSI). Shoot samples (0.1 g) were put in glass vials containing 10 mL of double-distilled water and the tubes were kept in water bath at 40 °C for 30 min. After cooling to 25 °C, the initial conductivity (C1) was recorded with an electrical conductivity meter (Model 335 D, Systronics, India). Subsequently, the same samples were placed in boiling water bath (100 °C) for 10 min and cooled to room temperature to record the final conductivity (C2). Membrane stability index (MSI) was calculated according to Premachandra et al. [73] as modified by Sairam [74].

MSI = [1 − (C1/C2)] × 100

Antioxidant enzyme activities. The activity of enzymes was determined in fresh tissue (shoots and roots) by spectrophotometric methods with the use of a double beam spectrophotometer U-2900 (Hitachi High-Technologies Corporation, Tokyo, Japan). Catalase (CAT) activity was measured according to the method described by Aebi [75] and peroxidase (POD) activity by Lück [76]. A total of 100 mg of plant material was homogenized in ice-cold conditions with 5 mL of pre-cooled 50 mM phosphate buffer with pH 7.0 for CAT and pH 6.2 for POD. The samples were centrifuged at 4 °C for 15 min at 4800× *g*. For analyses of CAT activity, 0.2 mL of extract, 1.8 mL of phosphate buffer were mixed with 1 mL of H_2_O_2_ solution in phosphate buffer. The absorbance (240 nm) of hydrogen peroxide decomposed by catalase was measured for 4 min in 1-min intervals. The unit of CAT activity was the amount of enzyme that decomposed 1µmol H2O2 in 1 min. For analyses of POD activity, p-phenyldiamine was used as enzyme substrate, which is oxidized by POD to phenazine. In total, 1 mL of extract, 1 mL of phosphate buffer, 0.1 mL of 1% solution of p phenyldiamine were mixed with 0.1 mL of 0.1% H_2_O_2_. The colored reaction product absorbance was measured at 485 nm. One unit of POD activity corresponds to an absorbance increase of 0.1.

Phenolic compounds estimation. The content of phenolic compounds was estimated using the photometric method with Folin’s reagent according to Singleton et al. [77] with slight modifications. Afterward,100 mg of plant material was homogenized with 4 mL of 80% methanol and centrifuged at 4 °C for 15 min at 4800× *g*. Extract was diluted 5 times with water. Subsequently, 1 mL of diluted extract was mixed with 0.2 mL of Folin–Ciocalteu phenol reagent (Sigma-Aldrich Chemie, GmBH, Steinheim, Germany) and 1.6 mL of 5% Na_2_CO_3_. The samples were incubated for 20 min at 40 °C. The absorbance of the mixtures was measured at 740 nm after cooling the samples. Chlorogenic acid was used as a reference standard and the results were expressed as milligram chlorogenic acid equivalents per 100 g of root or shoots fresh weight.

Total soluble carbohydrates (TSC). Total soluble carbohydrates (TSC) were measured according to Dubois et al. [78]. Dry material was ground and mixed with 3 mL of 80% methanol on a rocker shaker for 24–48 h. Sulphuric acid and 5% phenol were added and mixed before absorbance readings were taken at 490 nm. TSS contents were expressed as ‘mg equivalent of glucose’ per gram of DW.

Total soluble protein (TSP). For determining TSP, shoot samples (500 mg) were homogenized in 0.1 mL of phosphate buffer having pH of 7.5. The extract was centrifuged at 100,009× *g* for 20 min at −4 °C. The supernatant was used for the determination of protein and antioxidant enzyme assay. Total soluble protein was estimated following the method of Lowry et al. [79] using bovine serum albumin (BSA) as a standard.

Total free amino acid (TFAA). Total free amino acid (TFAA) content was estimated by the modified method of Colowick [80]. For this estimation, shoot sample (0.5 g) was homogenized in 80% ethanol (10 mL) and was then centrifuged at 8009× *g* in a refrigerated centrifuge for 10 min. Supernatant was taken, 0.1 N HCl (1 mL) was added, shaken and ninhydrin reagent (1 mL) was added. The mixture was heated for 20 min in a water bath. Water–propanol mixture (1:1) (5 mL) was then added and heated again in the water bath for 10 min. After cooling the test tubes, the absorbance was read at 570 nm on a UV-spectrophotometer.

Ions contents. Sodium and potassium concentrations in plant material was determined after dry combustion method and dissolving the ash in 1:3 H_2_O:HNO_3_ *v/v*. Double distilled water was used for extractions. Concentration of elements were determined using a Perkin Elmer atomic emission spectrometer ICP-OES Optima 7300 DV and multi-element ICP-IV Merck standard solution. The accuracy of the analytical methods was verified using GSS-8 certified reference material (GBW 07408; State Bureau of Meteorology, Beijing, China).

Each sample of the plant material was analyzed in two replications. If the analysis results of those replications differed from one another by more than ±5% another two analyses of that sample were conducted. Content of chloride in plant samples was determined in aqueous extracts, prepared by incubating 0.1 g of dried, ground shoot material in 15 mL of water for 1 h at 95 °C, and followed by filtration through a 0.80 μm filter. Chloride ion content was quantified using ion meter equipped with chloride ion selective electrode (9617BNWP Chloride Combination Electrode, Thermo Fisher Scientific, MA, USA).

### 4.5. Statistics

Every treatment was tested in two independent experiments, each with 7 replicates where 1 replicate was composed of two plants (two plants per pot, 14 plants per treatment.) Growth and biochemical data were processed by one-way ANOVA (*p* < 0.05). The significance of differences in growth and biochemical parameters between all experimental treatments was determined by Tukey’s and Student’s t test for *p* = 0.05. Quantitative data for biochemical compounds content were obtained from three biological replicates. Measured data are presented as the mean ± confidence interval for a 95% significance level.

A correlation matrix was applied to determine correlations for all biochemical parameters for 3 levels of NaCl for each variant. PCA was performed separately for each of the three variants of the experiment (I, II, III). Biochemical data were correlated with the level of applied salinity. Redundant analysis (RDA), a method of extracting and summarizing the variability in a set of response variables that can be explained with a set of explanatory variables, was also applied. Redundancy analysis was performed between salinity stress, molasses application and biochemical parameters. It was checked whether the addition of molasses changed the biochemical parameters within one salinity level. The statistics were created in Canoco for Windows version 4.5. The RDA can also be considered a limited version of principal component analysis (PCA) in which the canonical axes were constructed from linear combinations of response variables. Principal component analysis (PCA) was used to obtain a simultaneous matrix of observations and two-dimensional variables for all physiological parameters. The analysis was carried out in the PQ stat program, version 1.8.2. Pearson’s correlation was used to demonstrate linearity in all studies of physiological parameters. The linear correlation was performed in the corrplot package in the R 4.1 version.

## 5. Conclusions

The moderate level of soil salinity did not negatively affect the examined parameters of *T. serpyllum* under soil sugar beet molasses application, and even a content of K in plant tissues increased compared to control treatment. Soil application of 3% sugar beet molasses solution during the cultivation of wild thyme influenced stress tolerance index, stem growth, water content in roots, total soluble protein content or cell membrane integrity more positively than shoot irrigation. Although the physiological and biochemical basis for sugar beet molasses-induced tolerance is not clearly understood, we believe that a cascade of events is triggered to provide multiple stress tolerance in plants. Further research is warranted to elucidate the physiological and biochemical mechanisms by which molasses induces tolerance to salinity, and perhaps a variety of other abiotic environmental stresses.

## Figures and Tables

**Table 1:** Effect of sodium chloride and molasses on soil pH and soil EC after 5 weeks of plants treatment.

Treatments	pH	EC (dS m^−1^)
**I non SBM**		
0 mM NaCl	5.54 aA	0.98 aA
100 mM NaCl	6.08 bA	3.22 bA
200 mM NaCl	6.44 bA	4.64 cA
**II soil SBM**		
0 mM NaCl	5.38 aA	1.10 aA
100 mM NaCl	5.82 aA	3.34 aA
200 mM NaCl	6.34 aA	4.81±aA
**III foliar SBM**		
0 mM NaCl	5.46 aA	0.95 aA
100 mM NaCl	5.90 aA	3.14 aA
200 mM NaCl	6.40 aA	4.72 aA

The means ± SD (n = 3) are given in the table. For each variant of the experiment, lowercase letters indicate significant differences between NaCl level and different capital letters indicate significant differences between each variant experiment for the same NaCl level, according to Tukey’s test (α = 0.05); SBM—sugar beet molasses; soil SBM—sugar beet molasses applied to the soil; foliar SBM—foliar application of sugar beet molasses.

## Data Availability

Not applicable.
